# Rapid adaptive adjustments of selective attention following errors revealed by the time course of steady-state visual evoked potentials

**DOI:** 10.1016/j.neuroimage.2018.10.059

**Published:** 2019-02-01

**Authors:** Marco Steinhauser, Søren K. Andersen

**Affiliations:** a Department of Psychology, Catholic University of Eichstätt-Ingolstadt, Ostenstraße 25, 85072, Eichstätt, Germany; b School of Psychology, University of Aberdeen, Aberdeen, AB24 3FX, United Kingdom

**Keywords:** Cognitive control, Error monitoring, Event-related potentials, Selective attention, Steady-state visual evoked potentials

## Abstract

Directing attention to task-relevant stimuli is crucial for successful task performance, but too much attentional selectivity implies that new and unexpected information in the environment remains undetected. A possible mechanism for optimizing this fundamental trade-off could be an error monitoring system that immediately triggers attentional adjustments following the detection of behavioral errors. However, the existence of rapid adaptive post-error adjustments has been controversially debated. While preconscious error processing reflected by an error-related negativity (Ne/ERN) in the event-related potential has been shown to occur within milliseconds after errors, more recent studies concluded that error detection even impairs attentional selectivity and that adaptive adjustments are implemented, if at all, only after errors are consciously detected. Here, we employ steady-state visual evoked potentials elicited by continuously presented stimuli to precisely track the emergence of error-induced attentional adjustments. Our results indicate that errors lead to an immediate reallocation of attention towards task-relevant stimuli, which occurs simultaneously with the Ne/ERN. Single-trial variation of this adjustment was correlated with the Ne/ERN amplitude and predicted adaptive behavioral adjustments on the post-error trial. This suggests that early error monitoring in the medial frontal cortex is directly involved in eliciting adaptive attentional adjustments.

## Highlights

• SSVEPs were used to track the time course of attentional post-error adjustments.
• Errors lead to an immediate allocation of attention to relevant stimuli.
• Adjustments occur simultaneously with Ne/ERN and correlate with its amplitude.
• Adjustments predict adaptive behavioral adjustments on post-error trial.

## 1 Introduction

Successfully performing a difficult task requires that attention is directed to relevant information in the environment. This is however associated with a cost, as fully focusing on one task makes us less likely to notice information unrelated to that task. Attentional selection therefore comprises a fundamental trade-off. Too little selectivity may lead to poor task performance, but too much selectivity may lead to failure of detecting new and unexpected information. How does the brain find the right balance between too much and too little selectivity? Crucial for this ability might be an error monitoring system that continuously evaluates behavior and triggers adaptive adjustments of attention whenever necessary. As errors in tasks involving attentional selection often indicate insufficient attentional focus on task-relevant stimuli, error detection should elicit an immediate reallocation of selective attention to the relevant task, thus establishing a more optimal balance between attending task-relevant and task-irrelevant stimuli.

Studies using scalp EEG methods provided evidence for an error monitoring system in the medial frontal cortex (MFC) that generates an error signal already within 50 ms after an erroneous motor response (Holroyd and Coles, 2002; Yeung et al., 2004). While this MFC signal is also observed for unconscious errors, conscious error processing in widespread brain areas takes place between 200 and 500 ms post-error (Nieuwenhuis et al., 2001; Steinhauser and Yeung, 2010). These two stages of error processing are reflected by two distinct components in the EEG, the early error-related negativity (Ne/ERN; Falkenstein et al., 1990; Gehring et al., 1993) and the late error positivity (Pe; Nieuwenhuis et al., 2001; Steinhauser and Yeung, 2010). Studies on the Ne/ERN have shown that this error signal carries sufficient information for implementing adaptive attentional adjustments. It is sensitive to whether an error was caused by a task-irrelevant stimulus or not (Maier and Steinhauser, 2013) and correlates with the strength of post-error adjustments (Debener et al., 2005; Marco-Pallarés et al., 2008) and immediate error corrections (Gehring et al., 1993; Yeung et al., 2004). While this suggests that adjustments of attention could be implemented already within milliseconds after an error, there is no direct empirical evidence for such a rapid adaptive post-error adjustment elicited by the Ne/ERN.

Moreover, a controversy has emerged whether early attentional adjustments following errors are adaptive at all. While some studies demonstrated that post-error trials are associated with increased attention and task-related activity (Cohen and Van Gaal, 2013; Danielmeier et al., 2011; King et al., 2010; Maier et al., 2011; Steinhauser et al., 2017a, Steinhauser et al., 2017b), others found opposite results (Purcell and Kiani, 2016; Van der Borght et al., 2016), particularly when the interval between error and subsequent stimulus was short (Beatty et al., 2018; Buzzell et al., 2017). The latter supports the idea that error detection initially induces transient impairments of task processing due to capacity bottlenecks (Jentzsch and Dudschig, 2009), attentional orienting (Notebaert et al., 2009) or global suppression (Wessel and Aron, 2017) while adaptive adjustments are elicited at a later time point (Ullsperger and Danielmeier, 2016; Wessel, 2018). Such inertness of adaptive attentional adjustments would strongly limit the utility of error monitoring for balancing selectivity and optimizing performance.

In recent years, further insights into the interplay between error monitoring in the MFC and post-error adjustments have been provided by studies investigating oscillatory brain activity. Errors are typically followed by enhanced frontocentral theta oscillations as well as decreased posterior alpha oscillations (Cavanagh and Frank, 2014; Cohen and Van Gaal, 2013; Mazaheri et al., 2009; Navarro-Cebrian et al., 2016; van Driel et al., 2012). While the former is possibly related to the Ne/ERN and might represent error monitoring in the MFC, the latter could reflect post-error adjustments in arousal and attention. Indeed, both effects have been shown to be closely related (Cohen and Van Gaal, 2013; van Driel et al., 2012), and the error-related theta response is predictive of behavioral post-error adjustments (Valadez and Simons, 2018). However, the exact nature of the post-error alpha modulation is still unclear. While it could represent adaptive adjustments of attention and control (Cavanagh and Frank, 2014; Cohen and Van Gaal, 2013), it has frequently been discussed as a correlate of an unspecific arousal response (Navarro-Cebrian et al., 2013; Compton et al., 2018; Carp and Compton, 2009), possibly reflecting non-adaptive adjustments. Indeed, when errors were followed by another error, this was foreshadowed even by a stronger modulation of alpha activity (Cohen and Van Gaal, 2013). This demonstrates that more specific measures of selective attention are required to investigate whether error monitoring in the MFC leads to immediate adaptive attentional adjustments.

In the present study, we investigated the nature and precise time-course of attentional adjustments elicited by errors using steady-state visual evoked potentials (SSVEP). An SSVEP is the oscillatory brain response elicited by a flickering stimulus having the same frequency as the driving stimulus. SSVEP amplitude varies with the strength of attention directed to this stimulus and can be utilized to continuously measure the time-course of attentional allocation (Andersen and Müller, 2010; Kashiwase et al., 2012; Müller et al., 1998; Scherbaum et al., 2010). Here, we measured SSVEPs for task-relevant and task-irrelevant stimuli in a continuous global motion discrimination task (Andersen and Müller, 2010; Andersen et al., 2009) to precisely track the temporal dynamics of attention following errors and to determine the temporal relationship between attentional adjustments and the Ne/ERN. We hypothesized that, if the error monitoring system in the MFC directly initiates adaptive attentional adjustments, we should observe adaptive changes in the SSVEP immediately after the emergence of the Ne/ERN which vary with the size of the Ne/ERN amplitude. Given that adaptive adjustments following errors and conflicts have most frequently been shown to manifest as increased attention towards relevant stimuli (Danielmeier et al., 2011; Egner and Hirsch, 2005; King et al., 2010) rather than a suppression of irrelevant stimuli, we specifically expected to observe an adaptive reallocation of attention towards the relevant stimulus following errors.

## 2 Materials and methods

### 2.1 Participants

Seventeen participants (14 female) between 18 and 25 years of age (mean 22.0) with normal or corrected-to-normal vision and no history of neurological or psychiatric disorders participated in the experiment. No statistical methods were used to predetermine the sample size but our sample size was in a similar range as in previous studies using SSVEP techniques (Andersen et al., 2008, 2009; Kashiwase et al., 2012; Müller et al., 1998; Scherbaum et al., 2010) or similar experimental paradigms (Andersen et al., 2008; Scherbaum et al., 2010). All participants provided informed consent and received 8 Euro per hour or course credit. The study protocol was approved by the ethical committee of the Catholic University of Eichstätt-Ingolstadt.

### 2.2 Task and procedure

**Fig. 1:**
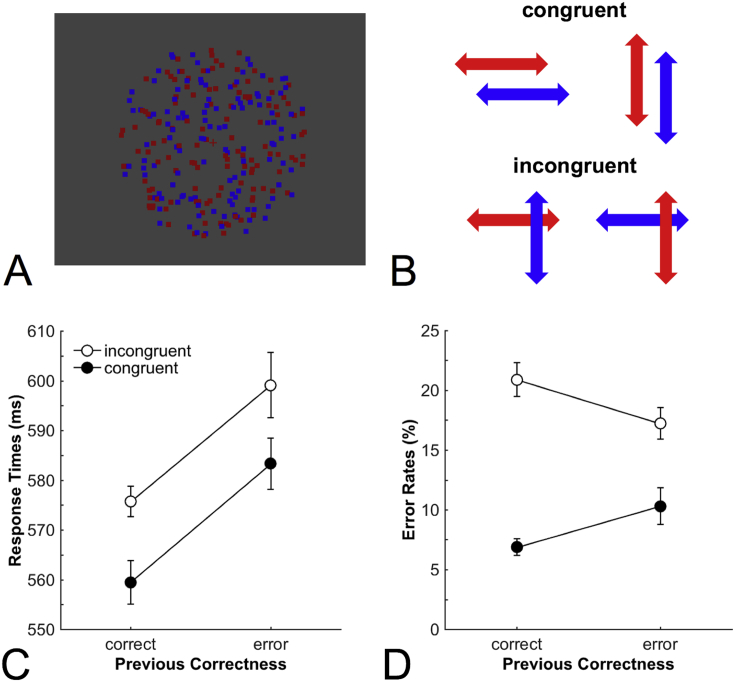
Experimental task and behavioral data. A: Exemplary stimulus display. In each run, participants viewed a continuous stream of two random-dot kinematograms (RDKs) consisting of randomly and incoherently moving red and blue dots. Embedded in each run were 20 trials in which both RDKs moved independently in one of the four cardinal directions for 500 ms. Participants had to indicate whether the relevant stimuli (indicated by the color of the fixation cross) moved horizontally or vertically by pressing a button. B: Congruent trials implied that both blue and red dots moved either horizontally or vertically. Incongruent trials implied that blue dots moved vertically and red dots moved horizontally, or vice versa. C: Mean response times of correct responses revealed a general slowing after error trials irrespective of whether the current trial was congruent or incongruent. D: Mean error rates showed a reduction of the congruency effect, and thus a focusing of attention to relevant stimuli, following error trials. Error bars represent within-subject standard errors of the mean. ms = milliseconds.

Our task was based on a global motion paradigm frequently used in SSVEP studies (e.g., Andersen and Müller, 2010), which we modified to make it more similar to the typical two-choice conflict paradigms used in the error monitoring literature. Stimulus displays consisted of two overlapping circular random dot kinematograms (RDKs) of red and blue color on a gray background (Fig. 1A), and were presented on a 21-inch cathode ray tube monitor with a resolution of 640 × 480 pixels and a refresh rate of 60 Hz at a viewing distance of 70 cm. RDKs flickered at a constant frequency linked to the color (red: 10 Hz; blue: 15 Hz). Each RDK consisted of 125 dots distributed over a circular area with a diameter of 14.17° visual angle. Each dot had a diameter of 0.37° and moved 0.12° in a random direction in every frame of screen refresh. Red and blue dots were drawn in random order to avoid systematic overlapping which could have induced a depth cue and were isoluminant to the gray background (4 cd/m^2^).

The task was to classify movements of the relevant stimulus (e.g., blue dots) while ignoring simultaneous movements of the irrelevant stimulus (e.g., red dots). Each trial consisted of a brief interval (500 ms) of coherent motion of the relevant and irrelevant stimuli in one of the four cardinal directions (up, down, left, or right). Coherence of movement was set to 75% to prevent tracking of individual dots. Relevant and irrelevant stimuli moved in independent directions. Participants had to indicate as fast as possible whether the relevant stimulus moved horizontally (left or right) or vertically (up or down) by pressing one of two response buttons with the index or middle finger of one hand. Hand and category-to-finger mapping was counterbalanced across participants. The movements of the relevant and irrelevant stimuli could be congruent (e.g., both moved horizontally) or incongruent (e.g., the relevant stimulus moved vertically, the irrelevant stimulus moved horizontally, see Fig. 1B).

The experiment consisted of 80 runs with 20 trials each, resulting in a total number of 1600 trials. A run started with a fixation cross of 1000 ms whose color indicated the relevant stimulus in this run. Then, the two RDKs were presented for 30 s while the fixation cross remained on the screen. The dots in both RDKs moved randomly and incoherently except for 20 uniformly distributed 500 ms coherent motion trials which were embedded in the continuous stimulation of each run. The minimum onset-to-onset separation between subsequent trials was 1200 ms. This resulted in a variable and potentially short interval between a response and the next movement onset (response-stimulus interval), which should promote immediate post-error adjustments. The interval between the end of each 30 s run and the next fixation cross was 1500 ms. After 10 runs, a feedback screen was presented that provided the proportion of misses and errors. A miss was defined as a trial for which no response occurred within 1000 ms after movement onset.

Prior to these experimental runs, participants worked through 24 practice runs. In contrast to the experimental runs, the feedback screen was presented after every 4 runs. During practice runs, participants received feedback on incorrect practice trials. If an incorrect response was provided, a 1500 Hz tone was immediately presented for 200 ms. If no response was provided within 1000 ms, a 600 Hz tone of the same length was presented.

### 2.3 Data acquisition

The electroencephalogram (EEG) was recorded using a BIOSEMI Active-Two system (BioSemi, Amsterdam, The Netherlands) with 64 Ag—AgCl electrodes from channels Fp1, AF7, AF3, F1, F3, F5, F7, FT7, FC5, FC3, FC1, C1, C3, C5, TP7, CP5, CP3, CP1, P1, P3, P5, P7, P9, PO7, PO3, O1, Iz, I1, I2, Oz, POz, Pz, CPz, Fpz, Fp2, AF8, AF4, AFz, Fz, F2, F4, F6, F8, FT8, FC6, FC4, FC2, FCz, Cz, C2, C4, C6, TP8, CP6, CP4, CP2, P2, P4, P6, P8, P10, PO8, PO4, O2 as well as the left and right mastoid. The CMS (Common Mode Sense) and DRL (Driven Right Leg) electrodes were used as reference and ground electrodes. Vertical and horizontal electrooculogram (EOG) was recorded from electrodes above and below the right eye and on the outer canthi of both eyes. All electrodes were off-line re-referenced to average reference. EEG and EOG data were continuously recorded at a sampling rate of 1024 Hz, and were offline re-sampled to 512 Hz. Data were collected in DC mode and no additional hardware filters were used during data acquisition, except for the in-built anti-aliasing filter of the amplifier.

### 2.4 Experimental design and statistical analysis

Each trial was assigned to a condition based on its Congruency (congruent, incongruent), the Relevant Stimulus (red, blue), and the post-hoc classification of Correctness (correct, error). The variable Relevant Stimulus was considered only during averaging to ensure that red and blue stimuli contributed equally to all averaged data.

#### 2.4.1 Behavioral data analysis

Trials for which no response had occurred between 200 and 1000 ms after stimulus onset (misses) were removed from this and all further analyses (m = 2.9%, s.e. = 0.4%). Response times (RT) of correct trials and error rates were considered to investigate post-error adjustments of performance, and hence, were analyzed as a function of Correctness of the previous trial and Congruency on the current trial. This and all further analyses were restricted to trials following trials with incongruent stimuli, because only incongruent trials produced a sufficient number of errors. A frequent problem with using correctness as a predictor is that, with a non-stationary error rate, correct trials and error trials (as well as post-correct and post-error trials) are not sampled equally from different parts of the experiment, thus confounding correctness with effects of global performance shifts (Dutilh et al., 2012). We therefore applied a two-stage averaging procedure: In a first stage, we averaged RTs and error rates separately for each run and condition (including the variable Relevant Stimulus). In a second stage, we averaged RTs and error rates for each condition across those runs for which both post-correct and post-error trials for a given condition were actually available (m = 41.8% of runs, s.e. = 2.4%). In this stage, data were collapsed across red and blue relevant stimuli. This procedure ensures that post-error and post-correct data from each run and each stimulus color contribute equally to the data. For the RT analysis, trials were excluded with RTs deviating more than three standard deviations from the mean computed for each condition and participant (<1%). Error rates were arcsine-transformed for statistical testing (Winer et al., 1991). All behavioral and EEG data subjected to parametric analyses (F-tests, t-tests) were tested for normality using Kolmogorov-Smirnov tests. No deviations from normality were detected.

#### 2.4.2 EEG data analysis

EEG data were analyzed using EEGLAB v12.0 (Delorme and Makeig, 2004) and custom routines written in Matlab 8 (The Mathworks, Natick, MA).^1^ Continuous EEG data were band-pass filtered excluding activity below 0.1 Hz and above 40 Hz. For the analysis of attentional adjustments on error trials, response-locked epochs were extracted ranging from 800 ms before and 1300 ms after the response. Channel-wise and epoch-wise artifact rejection was applied to all channels with the exception of the frontal channels AF7, Fp1, Fpz, Fp2, AF8 because eye blinks were corrected at a later stage. On average 1.18 channels per subject were interpolated using spherical spline interpolation because they met the joint probability criterion (threshold 5) and the kurtosis criterion (threshold 10) in EEGLAB's channel rejection routine (pop_rejchan.m). On average 13.8% of epochs were rejected because their amplitude fell above 200 μV or below −200 μV, because the joint probability of an epoch deviated more than five standard deviations from the distribution mean, or because horizontal or vertical eye movements were detected at the EOG channels. Blink artifacts were corrected by computing an independent component analysis (ICA) (Bell and Sejnowski, 1995) and removing blink components using CORRMAP 2.0 (Viola et al., 2009). Finally, misses and trials with error corrections (two or more responses, <1%) were removed from the analysis. To enable response-locked averaging without canceling out the steady-state response, we aligned response triggers with the phase of the stimulus flicker prior to averaging. Accordingly, two types of averaged waveforms were created for each condition: one in which triggers were shifted to the nearest onset of the 10 Hz stimulus, and one in which the same was done for the 15 Hz stimulus. Epochs were averaged separately for correct and error trials of incongruent stimuli using the same two-step procedure as described for the behavioral data. Moreover, to prevent that post-response activity was influenced by the subsequent stimulus, only trials with a response-stimulus interval larger than 350 ms were included (proportion of trials: m = 68.0%, s.e. = 2.9%). Altogether, this resulted in an average of 248.6 correct trials (s.e. = 12.6) and 59.2 error trials (s.e. = 7.1) included in the SSVEP analysis.

**Fig. 2:**
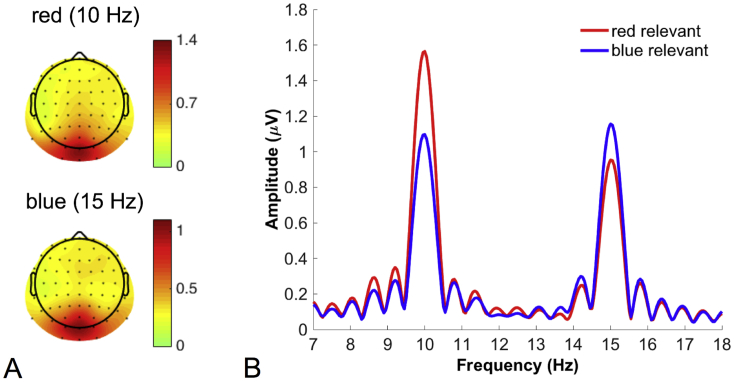
Amplitudes of steady-state visual evoked potentials (SSVEPs). A: Spatial distributions of SSVEP amplitudes at the two stimulated frequencies revealed the typical occipital distribution of the SSVEP signal. B: SSVEP amplitudes were enhanced at 10 Hz when the relevant stimulus was red but at 15 Hz when the relevant stimulus was blue.

To determine the appropriate electrode cluster for analysis, iso-contour voltage maps of the 10 Hz (blue) and 15 Hz (red) SSVEP amplitudes were calculated by means of Fourier transformation across whole epochs separately for runs in which participants had to attend to red and blue, respectively. Based on the spatial distribution of SSVEP amplitudes for each frequency (averaged across relevant stimuli, Fig. 2A) all further analyses were conducted using averaged data from channels POz, Oz, and Iz. The time course of base-to-peak SSVEP amplitudes was quantified by means of a Gabor filter (Gabor, 1946) at the respective center frequency. Both filters had an equal frequency resolution of ±1.60 Hz FWHM, resulting in a time resolution of ±138 ms FWHM. SSVEP amplitudes tend to decrease with increasing frequency, however relative attentional modulation has been found to be equivalent across a wide range of frequencies in previous experiments using similar stimuli (Andersen and Müller, 2010; Andersen et al., 2008, 2009). Following previous studies (Andersen and Müller, 2010), we analyzed the data separately for relevant and irrelevant stimuli in each condition (error, correct) while collapsing across stimuli (red, blue), and thus frequencies, after normalization (rescaling). Normalization was conducted by dividing the amplitudes for each data point by a reference amplitude for each frequency. As reference, we took the amplitude for relevant stimuli of the respective frequency, averaged across [-500; 500] relative to the response. The time course of attentional selectivity was computed by subtracting amplitudes for irrelevant stimuli from that of relevant stimuli.

Differences between correct and error trials at each sampling point were tested using two-tailed cluster-based permutation tests implemented in the Mass Univariate ERP Toolbox (Groppe et al., 2011), correcting for a family-wise alpha level of 0.05. After decimating the data to 10 Hz, clusters were formed by all time-points at which the uncorrected *p*-value was below 0.05. 10^5^ permutations were used to obtain the test distribution. To identify differences related to post-error adjustments, all time points between −50 ms and 350 ms relative to the response were included in the permutation test. The lower limit of this time window was chosen to capture the earliest time points at which error-related brain activity is typically obtained. To identify differences related to the source of errors, all time points between −500 ms and 0 ms relative to the response were included in the permutation test.

Error-related brain activity in event-related potentials was analyzed in response-locked epochs ranging from −200 ms to 350 ms relative to the response. To control for differences between errors and correct trials that emerge already during stimulus processing, activity in a time window of −150 to −50 ms relative to the response was taken as baseline (e.g., Steinhauser and Yeung, 2010). Artifacts were rejected and corrected as described above. The Ne/ERN amplitude was calculated as the difference in mean amplitude between correct and error trials at an electrode cluster around FCz (Cz, FC1, FCz, FC2, Fz) in a time window centered around the peak of the difference wave between correct and error trials (0–30 ms relative to the response). The Pe amplitude was calculated as the difference in mean amplitude between correct and error trials at an electrode cluster around Pz (POz, P1, Pz, P2, CPz) in the time window of 250–350 ms relative to the response (Steinhauser and Yeung, 2010).

To investigate the relationship between each of these components and post-error adjustment of relevant stimulus activity, single-trial SSVEP amplitudes for relevant and irrelevant stimuli were calculated by applying a Gabor filter to single-trial activity and projecting the obtained complex amplitudes onto their mean phase for each condition (Andersen et al., 2008). This procedure extracts evoked single-trial amplitudes by quantifying the contribution of single-trial activity to the phase-locked mean, thus reducing the influence of phase-unlocked noise. Error trials were classified as low-adjustment and high-adjustment trials based on a median split of SSVEP amplitudes of relevant stimuli in the time window of 100–200 ms after the response, i.e., the time window in which the post-error adjustment reached its maximum. Ne/ERN amplitudes were compared between these conditions. We used a median split instead of a regression-based approach because this ensures that the measured Ne/ERN amplitude within the low-adjustment and high-adjustment conditions still represents an evoked response (raw single-trial amplitudes confound phase-locked and phase-unlocked activity). To investigate whether the obtained relationship between Ne/ERN and relevant stimulus activity is specific for this time window, the analysis was repeated applying the median split of SSVEP amplitudes to a moving time window (width 10 ms, step size 10 ms) across the whole epoch. Statistical testing was done using the same approach as for the time-course analyses. The same analysis was repeated for irrelevant stimulus activity. Finally, to investigate the relationship between the strength of post-error adjustment of relevant stimulus activity and performance on the subsequent trial, mean RTs and error rates on post-error trials were analyzed as a function of the congruency on the current trial (congruent, incongruent) and the size of adjustment on the previous trial (low-adjustment, high-adjustment; again based on median split for SSVEP activity in 100–200 ms).

## 3 Results

### 3.1 Behavioral data

Our task required participants to categorize brief coherent movements of dots in one color (the relevant stimulus) while ignoring concurrent congruent or incongruent movements of dots in the other color (the irrelevant stimulus). We first analyzed behavioral data to investigate whether post-error adjustments of performance are observable in this task. The mean error rate was 9.7%. Mean RTs for errors (574 ms) and correct trials (567 ms) were not significantly different (*t*(16) = 1.22, *p* = .24, *d*_*z*_ = 0.30). To investigate whether errors led to behavioral adjustments on the subsequent trial, we subjected mean RTs of correct responses (Fig. 1C) and error rates (Fig. 1D) to a two-way repeated measurement ANOVA with the variables Congruency (congruent, incongruent) and Previous Correctness (correct, error). Mean RTs were higher on incongruent trials than on congruent trials (*F*(1, 16) = 6.01, *p* = .03, *η*_p_^2^ = 0.27), and were higher following errors than following correct responses (*F*(1, 16) = 15.9, *p* = .001, *η*_p_^2^ = 0.50), but there was no significant interaction (*F*(1, 16) < 0.01, *p* = .96, *η*_p_^2^ < 0.001). Mean error rates were higher for incongruent trials than for congruent trials (*F*(1, 16) = 37.5, *p* < .001, *η*_p_^2^ = 0.70), but this congruency effect was smaller following errors than following correct responses (*F*(1, 16) = 14.8, *p* < .001, *η*_p_^2^ = 0.48). Taken together, as in a previous study (Maier et al., 2011), we found post-error slowing in RTs but post-error reduction of interference in error rates. Whereas the former could be due to a more cautious response strategy, the latter indicates that errors were followed by enhanced attention to the relevant stimuli and/or reduced attention to the irrelevant stimuli.^2^

### 3.2 SSVEP data

Fig. 2A depicts the spatial distribution of SSVEP amplitudes at each flicker frequency averaged across all epochs. Because amplitudes peaked at channels POz, Oz, and Iz, all further analyses were conducted on average data from this cluster. Fig. 2B demonstrates that SSVEP amplitudes at this electrode cluster were modulated by attention. Amplitudes at 10 Hz (red RDK) were higher when red dots (relative to blue dots) were the relevant stimuli (*t*(16) = 7.23, *p* < .0001, *d*_*z*_ = 1.75), and amplitudes at 15 Hz (blue RDK) were higher when blue dots (relative to red dots) were the relevant stimuli (*t*(16) = 5.71, *p* < .0001, *d*_*z*_ = 1.38).

**Fig. 3:**
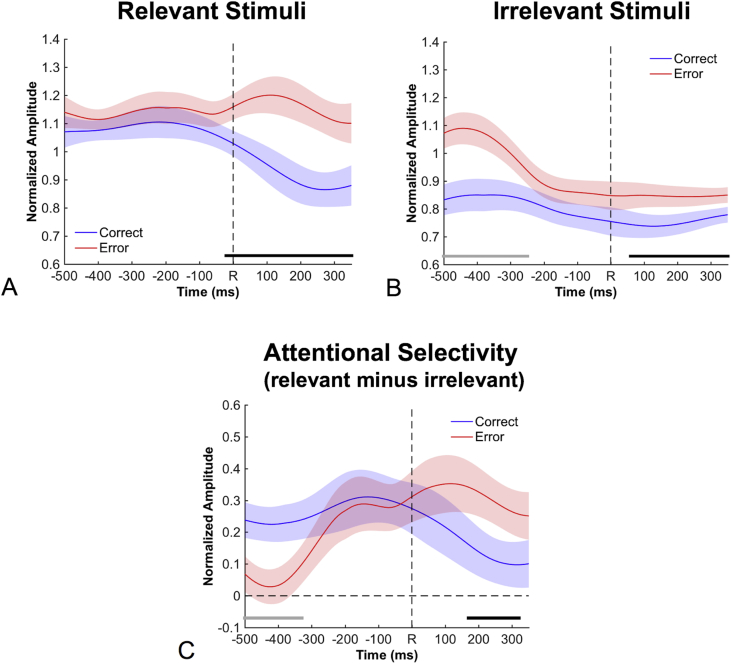
Time course of SSVEP amplitudes for relevant stimuli, irrelevant stimuli, and attentional selectivity. A: SSVEP amplitudes for relevant stimuli diverged significantly between errors and correct trials at 20 ms before the response, indicating a fast reallocation of attention to relevant stimuli on errors. B: SSVEP amplitudes for irrelevant stimuli were larger for errors than for correct trials across the whole epoch, although this difference reached significance only between −500 ms and −250 ms and later than 60 ms, presumably reflecting that errors predominantly occur when attention to irrelevant stimuli is increased. C: The net effect of these two patterns is represented by the difference between SSVEP amplitudes for relevant and irrelevant stimuli. The resulting measure of attentional selectivity is reduced before −340 ms and increased after 170 ms on errors relative to correct trials. Shaded areas reflect within-participant 95%-confidence intervals at the respective time point. Horizontal bars indicate significant differences between correct trials and errors in the pre-response phase (grey) and the post-response phase (black), as revealed by a cluster-based permutation test. ms = milliseconds.

The time course of the attentional modulation on error and correct trials was analyzed in two stages: Following Andersen and Müller (2010), we first considered SSVEP amplitudes separately for relevant and irrelevant incongruent stimuli in response-locked epochs.^3^ Activity for relevant and irrelevant stimuli was analyzed independently as attentional enhancement of relevant stimuli and suppression of irrelevant stimuli rely on distinct attentional mechanisms and follow different time courses (Andersen and Müller, 2010). In a second stage, attentional selectivity calculated as the difference between activity for relevant and irrelevant stimuli was compared between correct and error trials, which represents the interaction term of a Correctness by Relevance design. The data are depicted in Fig. 3. Horizontal bars in the figures represent significant differences between correct and error trials as revealed by a cluster-based permutation test (α = 5%, see methods for details). Amplitudes for irrelevant stimuli (Fig. 3B) were larger on error trials than on correct trials across the whole epoch. The difference reached significance between −500 and −250 ms in the pre-response period, and again starting at 60 ms after the response. This suggests that errors were primarily due to enhanced attention to irrelevant stimuli early in stimulus processing, and this enhanced attention presumably carried over to the post-response period. In contrast, amplitudes for relevant stimuli (Fig. 3A) were similar on correct and error trials before the response but then diverged around the time of response execution (with significant differences starting 20 ms before the response). This boost in amplitudes for relevant stimuli on error trials relative to correct trials reflects a surprisingly rapid adjustment of attention towards the target. Later, this rapid adjustment caused an increase in attentional selectivity (Fig. 3C), that is, in the difference between amplitudes to relevant and irrelevant stimuli, which was increased on error trials relative to correct trials starting at 170 ms after the response.

### 3.3 Relationship between error-related brain activity and SSVEP modulation

**Fig. 4:**
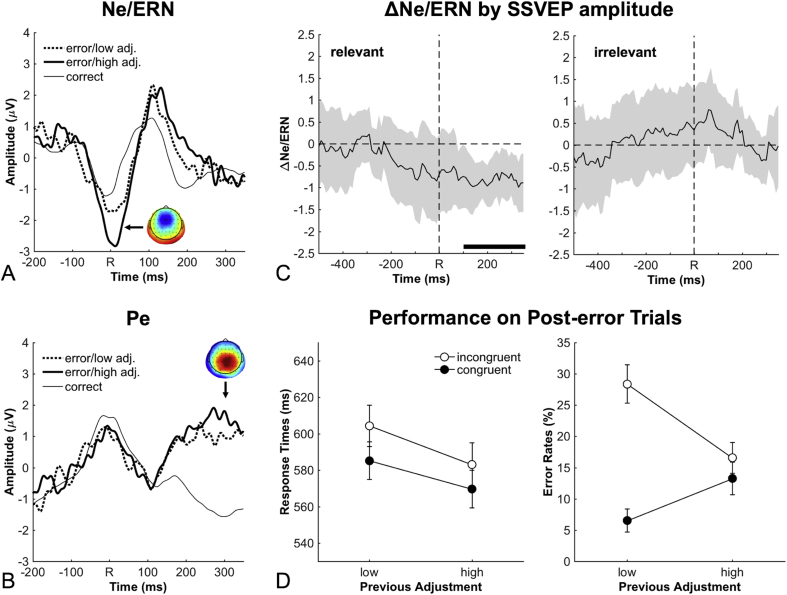
Error-related brain activity, post-error performance and their relationship with attentional adjustments in SSVEP amplitudes. A: Event-related potentials for correct and error trials revealed a larger Ne/ERN (0–30 ms, cluster around FCz) on high-adjustment trials than on low-adjustment trials. Error trials were categorized as high/low-adjustment based on a median split of SSVEP amplitudes for relevant stimuli between 100 and 200 ms (i.e., later than the time range of the Ne/ERN). Topography reflects the spatial distribution of the difference between errors and correct trials in the time range of the Ne/ERN. B: No comparable effect was obtained for the Pe (250–350 ms, cluster around Pz). Topography reflects the spatial distribution of the difference between errors and correct trials in the time range of the Pe. C: The Ne/ERN difference between high-adjustment and low-adjustment trials was recalculated for median splits on SSVEP amplitudes of relevant and irrelevant stimuli in consecutive time windows, thus creating a measure of the relationship between Ne/ERN and SSVEP signals over time. Black bars indicate significant time points in a cluster-based permutation test. The Ne/ERN was significantly related to SSVEP amplitudes of relevant stimuli starting at 110 ms, but unrelated to SSVEP amplitudes of irrelevant stimuli. Shaded areas reflect 95%-confidence intervals at the respective time point. D: To reveal how adjustments of SSVEP amplitudes for relevant stimuli affected performance on post-error trials, response times and error rates on post-error trials were calculated as a function of adjustment on the previous error trial (as defined above). Whereas no significant effect was obtained for response times, the congruency effect in the error rates was strongly reduced following high-adjustment error trials than following low-adjustment error trials. Error bars represent within-subject standard errors of the mean. ms = milliseconds.

Our results demonstrate that post-error adjustments of attention to relevant stimuli occur almost synchronously with the error response, and thus, at around the same time where early error-related brain activity is typically observed. In a further analysis, we therefore asked whether the strength of this adjustment is related to the size of the Ne/ERN. To this end, we compared Ne/ERN amplitudes between trials with high SSVEP amplitudes for relevant stimuli (high-adjustment trials) and trials with low SSVEP amplitudes for relevant stimuli (low-adjustment trials) in the time range where the mean adjustment reached its peak (100–200 ms after the response). Again, only incongruent trials were used in this analysis. Fig. 4A shows that Ne/ERN amplitudes were larger on high-adjustment trials than on low-adjustment trials (*t*(16) = 3.18, *p* < .006, *d*_*z*_ = 0.77). To investigate whether this effect was specific to this time period, and thus reflects a relationship between Ne/ERN and post-error adjustment, we repeated this analysis for consecutive time windows within our response-locked epoch. Fig. 4C (left panel) reveals that only in the time period of the post-error adjustment, amplitudes for relevant stimuli were predictive of the Ne/ERN with significant effects starting at 110 ms after the response. In contrast, the same analysis applied to amplitudes for irrelevant stimuli (Fig. 4C, right panel) revealed no effects at all. Specifically, in the above-mentioned time range, no significant Ne/ERN difference between trials with low SSVEP amplitudes for irrelevant stimuli and trials with high SSVEP amplitudes for irrelevant stimuli was obtained (*t*(16) = 0.53, *p* = .60, *d*_*z*_ = 0.13). We finally included the data for relevant and irrelevant stimuli in this time range in an ANOVA with the variables Stimulus (relevant, irrelevant) and Amplitudes (low, high), and obtained a significant interaction between both variables (*F*(1, 16) = 7.74, *p* = .01, *η*_p_^2^ = 0.33). These results are consistent with the idea that the Ne/ERN elicits a rapid post-error adjustment of attention to the relevant stimuli but not to the irrelevant stimuli.

In addition, we analyzed the later occurring Pe as a correlate of conscious error perception (Nieuwenhuis et al., 2001; Steinhauser and Yeung, 2010). As shown in Fig. 4B, the Pe did not differ between high-adjustment trials and low-adjustment trials (*t*(16) = 1.61, *p* = .13, *d*_*z*_ = 0.39). However, unlike the Ne/ERN, the Pe occurs *after* these adjustments, and thus, cannot reflect a causal influence of the Pe on these adjustments. These data merely show that the strength of post-error adjustment does not affect the emergence of the Pe.

### 3.4 Relationship between SSVEP modulation and post-error adjustments in behavior

Our behavioral data indicated two types of post-error adjustment: post-error slowing in RTs and a post-error reduction of interference in error rates. To investigate which of these behavioral adjustments is related to the observed adjustment of activity for relevant stimuli, we analyzed performance on trials following incongruent error trials. RTs and error rates from these post-error trials were submitted to a two-way ANOVA with the variables Congruency (congruent, incongruent) and Adjustment on Previous Trial (low-adjustment, high-adjustment). In the error rates (see Fig. 4D, right panel), we obtained a significant congruency effect (*F*(1, 16) = 14.2, *p* = .002, *η*_p_^2^ = 0.47), which was higher following low-adjustment trials than following high-adjustment trials (*F*(1, 16) = 12.4, *p* = .003, *η*_p_^2^ = 0.44). In fact, congruency had no significant effect at all following high-adjustment trials (*t*(16) = 0.78, *p* = .45, *d*_*z*_ = 0.19). In the RTs (see Fig. 4D, left panel), only a trend towards a lower mean RT following high-adjustment trials was revealed (*F*(1, 16) = 3.35, *p* = .09, *η*_p_^2^ = 0.17). This analysis demonstrates that the observed rapid adjustment of attention to relevant stimuli caused the post-error reduction of interference in the error rates as depicted in Fig. 1D. In contrast, post-error slowing appears to be unrelated to our SSVEP results since a higher post-error adjustment was even associated with a reduced post-error RT.

## 4 Discussion

**Fig. 5:**
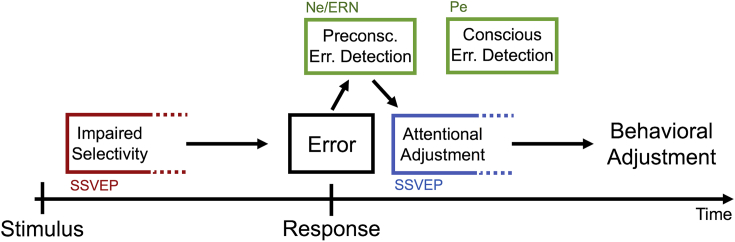
Summary of the sequence of events around errors. Each arrow represents a relationship revealed by our analyses. Impaired selectivity during stimulus processing in SSVEPs points to the origin of errors (red box). Errors are preconsciously detected as indicated by the Ne/ERN (left green box) which immediately leads to adaptive adjustments of attention (blue box) before conscious error processing reflected by the Pe takes place (right green box). Attentional adjustments finally lead to behavioral adjustments measured on the subsequent trial.

We tracked the time course of attentional adjustments following correct and error responses in a conflict task that required participants to discriminate the direction of coherent motion intervals embedded in continuous stimulation. A tightly linked cascade of events in the time before and after errors was observed (see Fig. 5). Error responses were preceded by a lack of attentional selectivity during processing of coherent-motion targets. An Ne/ERN occurred within 50 ms after the error response and the magnitude of this electrophysiological signature of preconscious error detection varied across trials with the strength of adaptive attentional adjustments reflected in an enhanced SSVEP to relevant stimuli in the first few hundred milliseconds after the error. The strength of these attentional adjustments in turn predicted performance adjustments on the subsequent trial. The temporal structure of this sequence of events is suggestive of a direct causal relationship: a lack of attentional selectivity causes errors whose rapid detection reflected in the Ne/ERN leads to almost immediate readjustment of attention which adaptively affects performance on the subsequent trial.

We propose that the post-error attentional adjustments reported here reflect a mechanism for adjusting the right balance between too little and too much selectivity and thus constitute a key determinant of the magnitude of attentional selectivity. This conclusion is based on two observations. First, attentional adjustments following errors were very large compared to the overall magnitude of attentional selectivity, which for this kind of stimuli and task has consistently been found to lie in the range corresponding to 30–50% enhancement of the attended stimulus relative to the unattended stimulus (Andersen and Müller, 2010; Andersen et al., 2008, 2009). In the present data, errors were preceded by an almost complete failure to selectively attend to the relevant stimulus, but in the initial hundreds of milliseconds after the error, selectivity was boosted to a level roughly corresponding to a 35–40% enhancement of the relevant stimulus (Fig. 3). Second, these attentional readjustments were highly effective in modulating subsequent performance. For those trials with the strongest boost of attentional selectivity, the behavioral interference of the distractor was almost entirely abolished on the subsequent trial (Fig. 4D). Conceptually, this pattern of enhanced selectivity following errors and slightly reduced selectivity following correct responses (Fig. 3C) is analogous to a psychophysical staircase technique, in which a physical stimulus parameter is adjusted to ensure a predetermined level of accuracy (Kaernbach, 1991; Watson and Pelli, 1983). In the present case, we propose that the brain adaptively adjusts internal attention parameters to enable a certain level of performance.

### 4.1 Implications for the functional role of error monitoring

Our data demonstrate that not only error processing in the MFC as reflected by the Ne/ERN but also the initiation of adaptive post-error adjustments occurs tremendously rapidly. This observation is in line with major accounts of error-related brain activity assuming that the Ne/ERN represents a signal that drives these adjustments. First, conflict monitoring theory (Yeung et al., 2004) assumes that the strength of adaptive adjustments is determined based on a post-response conflict reflected by the Ne/ERN. Second, the reinforcement learning theory of the Ne/ERN (Holroyd and Coles, 2002) proposed that the Ne/ERN is generated when a negative prediction error from the dopaminergic reward system reaches the MFC where adjustments are initiated. Both ideas receive direct support from the observed single-trial relation between the Ne/ERN amplitude and subsequent attentional reallocation. However, the temporal proximity of the Ne/ERN and attentional adjustments strongly suggests that the MFC not only signals the required strength of later implemented adjustments but directly triggers these adjustments.

Our results could implicate that conscious error perception is not necessary for post-error adjustments of attention to emerge. The Ne/ERN has been shown to be unrelated to error awareness. Even unaware errors can elicit an Ne/ERN (Wessel, 2012) and, under specific conditions, the Ne/ERN can be even negatively correlated with the level of error awareness (Di Gregorio et al., 2016). In contrast, there is evidence that the emergence of error awareness is reflected by the Pe (Nieuwenhuis et al., 2001; Steinhauser and Yeung, 2010), which has been shown to vary with the accumulated evidence for an error that underlies error awareness (Steinhauser and Yeung, 2010). Under the assumption that conscious error perception does not emerge before the time range of the Pe, the observation that attentional adjustments start and peak before the Pe suggests that these adjustments are triggered prior to the conscious perception of an error. However, such a conclusion can only be tentative as the specific mechanisms underlying error awareness are still under debate. Moreover, as we did not collect explicit measures of error awareness, no strong conclusions about the relationship of error awareness and the observed attentional adjustments can be drawn. It will be an interesting question for future studies whether error awareness can have a modulatory influence on adaptive attentional adjustments as measured in our paradigm.

### 4.2 Implications for the nature of post-error adjustments

The question emerges why we found only robust evidence for adaptive attentional adjustments given the increasing evidence from other studies that errors can have detrimental effects on attention, e.g., by eliciting an orienting response (Beatty et al., 2018; Buzzell et al., 2017; Notebaert et al., 2009; Van der Borght et al., 2016). First of all, variability of post-error adjustments across tasks could reflect that different tasks evoke different types of errors, and that post-error adjustments vary substantially depending on the error type (van Driel et al., 2012; Maier et al., 2011). For instance, O'Connell et al. (2009) used a time estimation task and found that SSVEP amplitudes did not predict subsequent errors, in contrast to what we observed in the present paradigm. Moreover, it is possible that non-adaptive adjustments are not reflected in SSVEPs in the immediate aftermath of an error but rather manifest at later time points, e.g., following the conscious perception of the error. Such a later occurring non-adaptive adjustment could be responsible for the observed post-error slowing, which was unrelated to the SSVEP modulation in our data (see Fig. 4D) and for the trend towards an increased error rate on post-error trials with short RSI. Future studies employing a stronger and systematic manipulation of RSI (e.g., as in Buzzell et al., 2017) in our paradigm could provide more evidence for this co-occurrence of adaptive and non-adaptive adjustments. A final possibility is that the attentional adjustment in our paradigm actually corresponds to the hypothesized orienting response (see also Murphy et al., 2016). It is conceivable that an error-induced orienting response actually serves to reallocate attention to goal-relevant stimuli. As stimuli are typically removed after the response in paradigms with discrete trials, such an orienting response could have detrimental effects in these studies. In contrast, under conditions of continuous stimulation - as in the present task - an orienting response towards the target is highly adaptive. From this view, an intriguing prediction can be derived which can be tested in future research: Whether adaptive or non-adaptive post-error adjustments are obtained might depend on whether goal-relevant stimuli, to which attention can be directed, are displayed between trials.

We found that the Ne/ERN amplitude was associated with the strength of post-error adjustments (Debener et al., 2005; Marco-Pallarés et al., 2008). Crucially, whereas activity for both relevant and irrelevant stimuli was increased following errors (as compared to following correct responses), only the enhancement of relevant stimuli correlated with the Ne/ERN. This additionally supports our conclusion that error monitoring elicits only an adjustment of activity to relevant stimuli whereas any effects on activity to irrelevant stimuli in our data reflect the source of an error. Moreover, this finding is in accord with biased-competition accounts (Desimone and Duncan, 1995) suggesting that top-down control of selective attention is achieved by biasing attention to the target which can later lead to distractor suppression by means of local competition (Andersen and Müller, 2010). This could explain why previous studies more consistently reported enhanced task-related activity rather than suppression of task-unrelated activity in post-error trials (Danielmeier et al., 2011; King et al., 2010) and post-conflict trials (Egner and Hirsch, 2005). However, these studies reported adjustments in later category-specific areas (like the fusiform face area for face stimuli) whereas the present effects occurred much earlier in the visual stream. SSVEP attention effects with the present stimuli have been localized in the initial stages of visual processing (V1—V3) (Andersen and Müller, 2010; Andersen et al., 2008, 2009). Thus, the attentional adjustments following error responses observed here are not just temporally but also anatomically early and reflect enhanced processing of relevant stimuli already at the initial stages of cortical processing.

We chose 10 and 15 Hz as tag frequencies in the present study because these frequencies have previously yielded robust SSVEPs with similar stimuli (e.g., Andersen et al., 2011), are sufficiently separated to allow for good temporal resolution without cross-talk, and synchronize frequently (every 200 ms). Visual stimulation at 10 Hz has recently been reported to entrain endogenous alpha rhythms and thereby affect performance (Spaak et al., 2014; Gulbinaite et al., 2017). This raises the question whether the present results could reflect an interaction between alpha entrainment and post-error decrease of alpha power (Mazaheri et al., 2009; van Driel et al., 2012; Cohen and Van Gaal, 2013). If this were the case, our results should differ qualitatively between the 10 and 15 Hz stimuli, however errors were followed by an enhanced SSVEP to relevant stimuli for both 10 and 15 Hz. This is consistent with a range of previous studies using similar stimuli that have generally observed equivalent attention effects across flicker frequencies (e.g., Andersen et al., 2008, 2011). This absence of frequency specific attention effects is possible because such effects ‘may be difficult to uncover if the relationship between exogenous rhythm (flicker) and endogenous rhythms (neural oscillations) is not taken into consideration.’ (Gulbinaite et al., 2017).

### 4.3 Conclusions

To summarize, the present study provides evidence that errors elicit a rapid adaptive reallocation of attention to relevant stimuli. This post-error adjustment occurs at the time of the Ne/ERN and varies with its amplitude, suggesting that it is directly triggered by early error processing in the MFC. Our results thus demonstrate a fast and flexible interplay between error monitoring and selective attention in the human brain. Given the growing evidence that various psychiatric and neurological diseases are associated with altered error monitoring in the MFC (Weinberg et al., 2015), our findings raise the possibility that these error monitoring deficits could underlie pathological decrements in attentional adaptiveness.

## Funding

This work was supported by a grant from the Biotechnology and Biological Sciences Research Council (BB/P002404/1 to S.K.A.).

## Footnotes

1 Data and customized analysis scripts can be found at https://osf.io/v6rpz.

2 Buzzell et al. (2017) have shown that adjustments on post-error trials vary with response-stimulus-interval (RSI), i.e., the time between error and subsequent stimulus. We therefore investigated how the above-mentioned effects were affected by RSI. Separately for each condition, we conducted a median split over RSIs (preceding the post-error trial) and included the variable RSI (short, long) in the ANOVAs. The average short and long RSI across conditions was 339 ms (s.e. = 5.4 ms) and 605 ms (s.e. = 6.4 ms), respectively. The only effect involving RSI and Correctness was a marginally significant interaction between RSI and Previous Correctness in the error rates (*F*(1, 16) = 3.13, *p* = .096, *η*_p_^2^ = 0.16), which resembles the pattern in Buzzell et al. (2017). There was a post-error decrease of the error rate by 1.63% (s.e. = 1.30%) with long RSIs and a post-error increase of the error rate by 2.79% (s.e. = 1.30%) with short RSIs. However, none of the adaptive post-error adjustments reported in the main analysis interacted significantly with RSI. Crucially, we found the same significant effects for short and long RSIs, i.e., post-error reduction of interference in the error rates (short: *F*(1, 16) = 10.0, *p* = .006, *η*_p_^2^ = 0.39; long: *F*(1, 16) = 6.94, *p* = .02, *η*_p_^2^ = 0.30) and post-error slowing in the RTs (short: *F*(1, 16) = 10.1, *p* = .006, *η*_p_^2^ = 0.39; long: *F*(1, 16) = 6.59, *p* = .02, *η*_p_^2^ = 0.29).

3 All data are presented averaged across frequencies after normalization (see methods). However, key results like the attentional modulation after errors were also obtained when data for 10 Hz and 15 Hz frequencies were analyzed separately.
